# Virtual reality for activities of daily living rehabilitation after acquired brain injury

**DOI:** 10.3389/fresc.2026.1726559

**Published:** 2026-06-30

**Authors:** Jasleen Grewal, Alyssa Turcott, Jodi Ferrer, Janice J. Eng, Brodie Sakakibara, Julia Schmidt

**Affiliations:** 1 Rehabilitation Science Graduate Program, University of British Columbia, Vancouver, Canada; 2 Rehabilitation Research Program, Centre for Aging SMART at Vancouver Coastal Health, Vancouver, Canada; 3 Department of Occupational Science and Occupational Therapy, University of British Columbia, Vancouver, Canada; 4 Department of Physical Therapy, University of British Columbia, Vancouver, Canada; 5 Centre for Chronic Disease Prevention and Management, University of British Columbia, Kelowna, Canada

**Keywords:** acquired brain injury, activities of daily living, occupational therapy, technology, virtual reality

## Abstract

**Background:**

Acquired brain injury (ABI) can result in reduced activities of daily living (ADL) performance. Virtual reality (VR) is a novel rehabilitation intervention for ABI. The Saebo-VR is a non-immersive platform for practicing ADL tasks.

**Objectives:**

To explore the implementation of a VR-based intervention for ADLs to improve function after ABI.

**Materials and methods:**

A pre- and post-intervention study was conducted using the Reach, Effectiveness, Adoption, Implementation, and Maintenance (RE-AIM) framework. Data from both groups were used to evaluate all domains of the RE-AIM framework. Participants with ABI completed 12 30-minute Saebo-VR sessions over 3-weeks to practice ADLs. The Saebo-VR is a non-immersive VR device programmed with ADLs.

**Results:**

Fifteen participants (mean age 46.5, 10 male) with ABI and 5 occupational therapists (mean age 37.8, all female) participated. For reach, 23% people with ABI participated. For effectiveness, a linear mixed-effects model was conducted to assess changes in Nottingham Extended Activities of Daily Living (NADL) scores from baseline to post-intervention. There was a statistically significant improvement in NADL scores [*b* = 9.4, SE = 3.1, *t*(23.9) = 3.1, *p* = 0.005, 95% CI (3.4, 15.4)], exceeding the MCID. For adoption, perceived benefits and challenges respectively included benefits to physical function and technical issues and negative impacts. Implementation indicators were partially met. For maintenance, only 60% of clinicians reported they would use VR clinically in the future.

**Conclusions and significance:**

Exploring the implementation of VR interventions with the ABI population provides key information on the clinical use of VR.

## Introduction

Acquired brain injury (ABI) is an umbrella term which refers to traumatic or nontraumatic damage to the brain occurring after birth, including stroke and traumatic brain injury (1). Globally, moderate-to-severe traumatic brain injury (hereafter referred to as TBI) and stroke respectively impact approximately 13.1 and 16.9 million people per year (2, 3). When a person sustains an ABI, their overall health and well-being are impacted due to experiencing a range of impairments. These impairments include physical challenges, such as reduced range of motion or balance (4, 5), cognitive challenges, such as difficulty with memory or attention (6–8), sensory concerns, such as spatial neglect (9, 10), and difficulties with emotional function, including depression or anxiety (11, 12). These impairments typically emerge after injury and often continue long-term impacting a person over the course of many years (13).

After sustaining an ABI, people often experience challenges reengaging in their lives due to difficulties completing their activities of daily living (ADL) (14–16). The ability to complete ADLs requires physical, cognitive, sensory, and emotional functioning (17, 18). Therefore, some people with ABI who experience deficits in these domains may experience challenges in completing ADLs, depending on the severity and context of their impairments (16, 19–21). Reduced capacity in completing ADLs may result in increased length of stay in the hospital (22, 23), additional reliance on caregivers (24), and reduced independence (25, 26). As such, improving ability to perform ADLs is a key focus of rehabilitation. People with ABI may engage in rehabilitation to maintain, restore, or enhance functioning, including ADL performance. Specifically, after ABI, occupational therapists (OT), as part of an interdisciplinary rehabilitation team, help improve ADL performance through targeted assessments and interventions in hospital, clinic, and home settings (27). Research shows that interventions for ADL function vary across rehabilitation settings (28). Often, in hospital settings, interventions focus on improving physical function (i.e., upper extremity strength and range of motion) and cognitive skills (i.e., memory, attention, sequencing) (29, 30). Although these interventions may enhance ADL function, research suggests that ADL rehabilitation is complex, and that no single intervention or approach has been proven to be more effective at enhancing ADLs (28, 31).

In recent years, there has been a growing interest in using virtual reality (VR) for rehabilitation. VR is defined as a three-dimensional computer-generated environment displaying digitally delivered simulations of real world or artificial environments (32). VR environments can also provide feedback through different senses, such as hearing, touch, and visual feedback. VR devices vary in immersion, which is the extent to which a user perceives they are in the virtual environment as opposed to the physical environment or the technological capability of the system (33, 34). The level of immersion in a VR system can be understood along a continuum from non-immersive to fully immersive (35). Non-immersive systems allow users to remain primarily in control of their real environment, semi-immersive systems enable partial interaction with simulated environments, and fully immersive systems use head-mounted displays to create a sense of physical presence in virtual environments (34, 36). In addition to VR, other types of digital therapies, such as mixed reality and augmented reality, are emerging in ABI rehabilitation. Mixed reality or augmented reality may be better tolerated than fully immersive VR in some ABI populations due to lower cognitive and sensory demands.

In rehabilitation, VR can facilitate people to practice simulated functional tasks at a higher dose than traditional therapies (37, 38). Some VR devices are programmed with features, such as the ability to generate reports based on the person's performance (37, 38). VR also allows repetitive task training, which may improve walking distance and speed, and upper extremity function (39, 40). VR within a rehabilitation context can be useful to practice tasks that which would be unsafe to complete in the real world, such as crossing a street (41). As VR provides additional sensory cues and engagement, it may be an interesting and motivating platform for some (42), therefore encouraging more repetitive task training and supporting principles of motor learning, neuroplasticity, and skill generalization, which may enhance occupational performance (38, 43–44). Digital therapies like mixed and augmented reality may also enhance task repetition and functional outcomes while potentially improving clinical efficiency and resource use (45, 46).

There has been past research demonstrating that VR may be promising for rehabilitation. For example, a Cochrane review showed that VR can improve upper limb function and daily activities, showing a small but greater benefit compared to alternative therapies (47). VR may also be a safe intervention that has potential to improve upper extremity and ADL function after stroke, however this improvement may only occur at a higher dose (i.e., when combined with usual therapy) and is not statistically significant (47). Research also shows that VR interventions incorporating ADLs are limited in the ABI population and demonstrate mixed results (48). For instance, a study showed that VR interventions incorporating ADLs (e.g., Saebo-VR) can improve upper extremity function (49). However, a randomized control trial (RCT) showed that there were no statistically significant improvements in performing daily activities after a VR intervention incorporating ADLs (50). In contrast, a pre- and post-intervention study reported statistically significant improvements in performing daily activities after a VR intervention (51). Yet, another pre- and post-intervention study indicated slight improvements in some daily activities (52). The level of immersion may also influence the effectiveness of VR. Research shows that fully immersive VR devices may be the most effective, producing the largest gains in Fugl-Meyer Assessment scores, while semi-immersive VR may yield smaller improvements and non-immersive VR provides intermediate benefits (53). Given that VR interventions incorporating ADLs have demonstrated mixed effects on functional outcomes, there is a need to examine their implementation in clinical practice. The Reach, Effectiveness, Adoption, Implementation, Maintenance (RE-AIM) framework can evaluate how VR interventions can be effectively integrated into rehabilitation programs for people with ABI (54). Therefore, our primary aim was to understand the implementation of a VR intervention incorporating ADLs in people with ABI using a pre- and post-intervention study design guided by the RE-AIM framework.

## Methods

### Study design

We conducted a pre- and post-intervention study using the RE-AIM framework. The RE-AIM framework provided a systematic and comprehensive framework to assess the implementation of the VR intervention (54). Specifically, reach refers to the number or representativeness of people who take part in the intervention; effectiveness is the extent to which the intervention produced a beneficial effect on the outcomes; adoption reflected the satisfaction and challenges participants experienced while receiving or delivering the intervention (55); implementation is the degree to which the intervention is delivered as intended; and maintenance is the degree to which an intervention is integrated into the organization's usual practices (56, 57).

Data were collected in-person at GF Strong Rehabilitation Centre or online via a secured platform (i.e., secured Zoom) from April 2024 to January 2025. We obtained ethics approval from the University of British Columbia (#H24-00444). Results are reported using the Standards for Reporting Implementation Studies (StaRI) checklist (Supplementary Appendix A) (58).

### Participants

#### Participants with ABI

Purposeful sampling was used to recruit participants with ABI from inpatient and outpatient settings at GF Strong Rehabilitation Centre. Participants were recruited through an OT working in the inpatient unit or through study advertisements. Participants were screened by OTs, researchers with an occupational therapy background (JG and JS), study coordinators, or a research assistant (AT). The inclusion criteria were: (1) diagnosis of moderate-to-severe stroke or TBI, (2) be within 1-year of diagnosis of stroke or TBI, (3) aged between 19- to 70-years, and (4) able to communicate in English. Injury severity was determined by level of impairment based on clinical judgement (i.e., only patients with severe injuries were admitted to the rehabilitation centre) and the Glasgow Coma Scale at the time of injury (latter only for participants with TBI) (59). The exclusion criteria were: (1) diagnosis of mild stroke or TBI, (2) ABI due to other aetiologies, such as anoxic/hypoxic injury, brain tumour, infection, or other neurological conditions, (3) diagnosis of receptive or expressive aphasia, (4) having a flaccid upper extremity, and (5) substantial health conditions (dementia, epilepsy, substantial visual impairment, and/or judged by a clinician to be too sick to take part in the VR intervention). Only people with moderate-to-severe stroke or TBI were recruited, as these represent the most common ABI aetiologies and share key characteristics, including similar functional consequences and rehabilitation needs (60–62). The sample size of ABI participants (*n* = 15) was informed by previous pre- and post-intervention studies implementing VR with the ABI population (49, 51) and previous studies with similar methodology (55, 63). Most studies in a scoping review showed that recruitment of ABI participants varied from 10 to 20 participants, with an average of 13.6 participants across pre- and post-intervention design studies (48). Some of these studies demonstrated significant improvements in the outcome measures based on this sample size (49, 51, 64). Consent to participate was collected in-person after participants were screened.

#### Clinician participants

Clinician participants were recruited from inpatient and outpatient ABI programs from GF Strong Rehabilitation Centre. Clinicians were recruited through word of mouth or study advertisements. Clinicians were recruited if they were a registered OT working in ABI rehabilitation at a rehabilitation facility or had experience working with the ABI population. Consent to participate was collected in-person by researchers.

### VR intervention and device

Participants were recruited over 6-months at GF Strong Rehabilitation Centre. After obtaining consent, ABI participants completed up to 12 VR sessions (30-min each) over 3-weeks. The VR sessions were facilitated by OTs, researcher with an occupational therapy background (JG), or research assistants (AT and JF) with an observer present to complete a fidelity checklist. All members of the research team underwent a 1-h training conducted by JG on how to facilitate the sessions and complete the fidelity checklist. Clinicians were offered to attend up to two 1-h training sessions that emphasized the purpose of the intervention sessions (i.e., practicing ADLs). ABI participants continued to receive standard occupational therapy [approximately three to four sessions (60-min) per week] and other relevant rehabilitation [approximately three to four sessions (60-min) of each discipline per week] during their involvement in the VR study.

**Figure 1:**
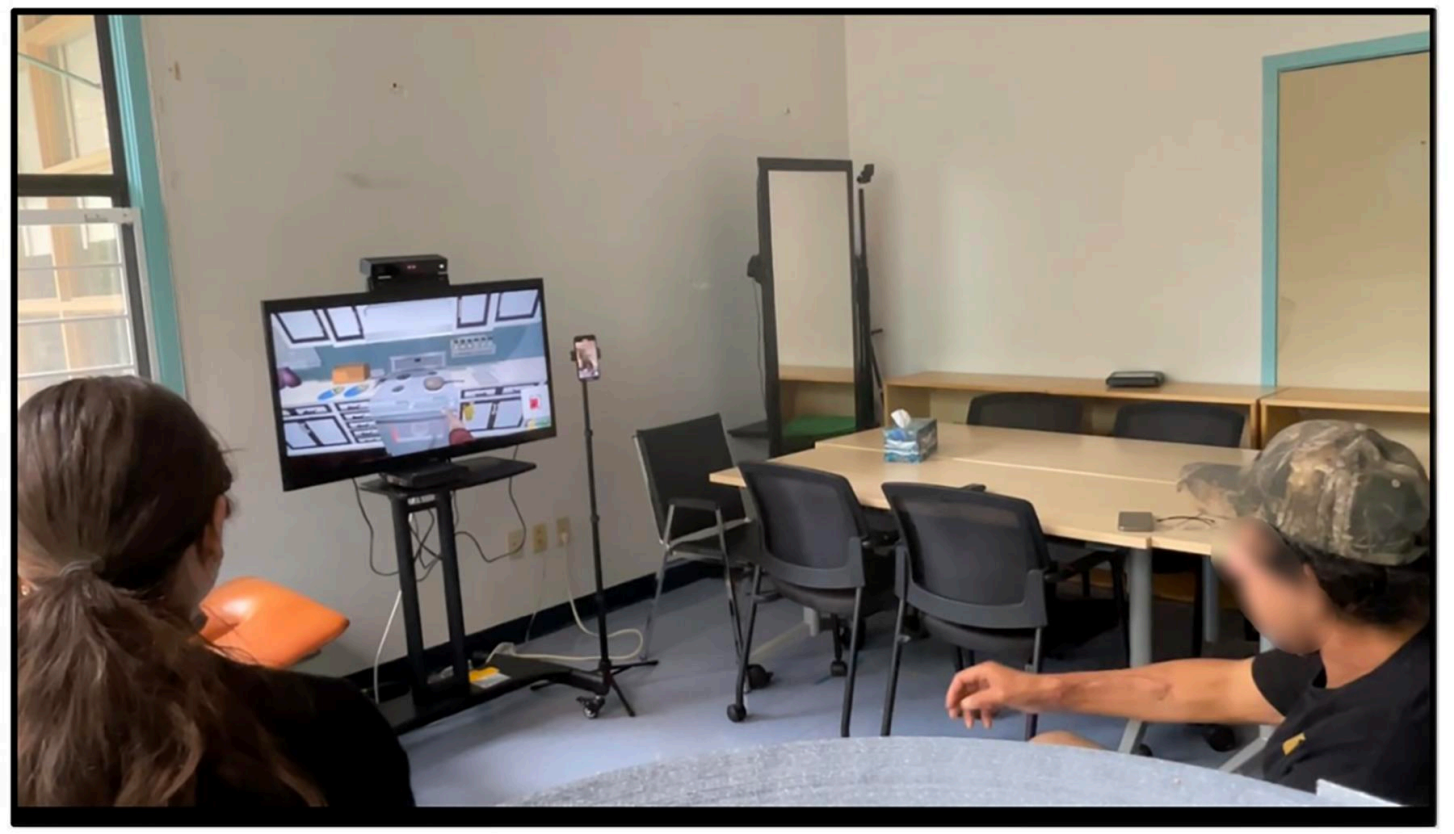
Saebo-VR setup with a participant with ABI and facilitator.

The Saebo-VR, a non-immersive VR device, was used for the intervention. The Saebo-VR consists of a monitor, Microsoft Kinect camera, a keyboard, and remote control (65). The Kinect camera tracked gross motor (i.e., shoulder and elbow extension/flexion) movements of one upper extremity. The monitor displayed the virtual environment and the simulated upper extremity. The device included a balls and box activity and 11 daily tasks (65). For our study, we had participants complete the grocery shopping, putting away groceries, pet feeding, making breakfast, making dinner, organizing closet, and soup kitchen tasks. We excluded other tasks as they may not have been applicable to participants' daily lives (i.e., garden harvesting). Tasks like pet feeding or soup kitchen activities were chosen for their focus on food preparation, similar to other tasks such as breakfast or dinner making, ensuring participants practiced the targeted motor and cognitive skills even if the context was not personally familiar. The keyboard was used by facilitators to choose the activities, ensure the Microsoft Kinect camera was tracking the participant's upper extremity, and complete automatic completions of tasks (i.e., overriding the task if participants had difficulty completing it due to technology concerns or upper extremity limitations). The remote control allowed the facilitator to turn on the monitor. The Saebo-VR was positioned approximately 5-metres away from participants to ensure accurate tracking of the upper extremity (Figure 1).

All session facilitators (i.e., researchers and clinicians) were instructed on operating the Saebo-VR, including key functions, such as ensuring the Microsoft Kinect camera was tracking the participant's upper extremity, completing automatic task completions, and troubleshooting technical issues. They were also given the opportunity to practice using the device with research staff and each other. To further support clinicians, during intervention delivery, research staff was present to support using the Saebo-VR and manage any technical difficulties.

### Data collection

Data were collected at three time points: pre-intervention, post-intervention, and at 3-months post-intervention. Data were used to address the primary aim of evaluating the implementation of a VR intervention incorporating ADLs for individuals with ABI.

#### RE-AIM data sources

For reach, key demographic and clinical factors (i.e., age, sex, days since injury, years of clinical experience, etc.) were collected during participant screening and on the pre-intervention demographics form.

Effectiveness was assessed within the RE-AIM framework using outcome measures at pre-intervention, post-intervention, and 3-month follow-up. The primary outcome for the quantitative analysis was the Nottingham Extended ADL Scale (NADL), and the secondary outcome measures included the Fugl-Myer Upper Extremity Scale (FMUE) (66), Montreal Cognitive Assessment (MOCA) (67), and the Daily Living Self-Efficacy Scale (DLSES) (68) (Supplementary Appendix B). The NADL is a self-report measure that assesses function in 22 activities across four categories: mobility, kitchen, domestic, and leisure (69). The scoring varies from 0 to 3, with a maximum score of 66 (70, 71). Within the effectiveness component of the RE-AIM framework, the primary analysis focused on whether there was a significant change in NADL scores from pre- to post-intervention. Additional analyses examined changes in the other outcome measures from pre- to post-intervention and whether any observed changes were maintained at the 3-month follow-up.

For adoption, post-intervention semi-structured interviews were conducted with participants with ABI and OTs to explore perceived facilitators and barriers to VR adoption in clinical practice. Interviews were completed following the intervention period (Supplementary Appendix C) and audio-recorded with participant consent.

For implementation, the fidelity checklist included session length (i.e., set up time, time spent completing ADLs, breaks), VR practice length (i.e., time spent completing VR tasks), length of breaks, reasoning for why a session ended early, assistance provided, technical issues, the number of verbal cues, encouragement provided, or negative impacts experienced by the participant. Three out of 12 sessions (30%) were also video recorded to ensure fidelity of the intervention, specifically the researchers watched the video recordings to ensure the intervention was delivered consistently.

For maintenance, data were collected at the 3-month follow-up. Participants with ABI were interviewed in-person or via Zoom and asked whether they would be willing to use the VR intervention in rehabilitation again, and to explain their reasons. Clinicians provided electronic written responses indicating whether they would be willing to continue using or implementing the VR device in rehabilitation long-term, and to describe their reasons (Table 1).

**Table 1:** RE-AIM measures and data sources.

Assessment level	Measures	Data Sources	Timeline
Reach	Recruitment rate	% of participants approached who were eligible and agreed to participate	Pre-intervention
	Participant demographics	Demographics form	
Effectiveness	Treatment efficacy	Outcome measure assessments	Baseline, post-intervention, and 3-month follow-up
Adoption	Perceived benefits and challenges of the intervention	Participant (clinician and people with ABI) interviews	Post-intervention
Implementation	Fidelity of intervention, attendance rate, adaptations made	Fidelity checklist, video recordings of intervention sessions	During intervention, post-intervention
Maintenance	Extent to which the intervention is intended to be sustained over time	Whether participants are willing to use the VR device long-term	3-month follow-up

### Procedure

The VR sessions occurred at GF Strong Rehabilitation Centre. At the beginning of each session, the facilitator ensured the Microsoft Kinect camera was tracking the participant's upper extremity in a seated or standing position. Then, participants completed the balls and box task twice to ensure they were accustomed to the upper extremity movements required by the Saebo-VR. After this, the participant completed as many daily tasks as they could tolerate during the 30-minute session. The facilitators offered breaks and provided verbal cues as needed. The facilitators could assist the participant by overriding a task using automatic completions or completing the task for the participant. Facilitators also encouraged the participant. An observer was present during sessions to complete the fidelity checklist. After the last intervention session, participants completed a semi-structured interview. Clinicians also completed interviews after the last session they facilitated. At the 3-month follow-up, participants with ABI completed the outcome measures and all participants were asked if they would be willing to use the VR intervention long-term.

### Data analysis

Descriptive statistics were used to analyze data for reach of the RE-AIM framework. For effectiveness, descriptive statistics were used to summarize the primary outcome measure, including baseline and post-intervention means, mean change, and effect sizes (Cohen's *d*) to illustrate the magnitude of change. Subgroup analyses were conducted by sex, diagnosis, care setting (inpatient vs. outpatient), and days post-injury, with mean change in NADL scores reported for each subgroup. Subgroup analyses were treated as exploratory due to the small and imbalanced sample across subgroups. Linear mixed-effects models (LMM) were used to analyze changes in all outcome measures from pre- to post-intervention. Time (pre-, post-intervention, 3-months post-intervention) was included as a fixed effect, and a random intercept was specified for each participant to account for within-participant correlations. Missing data were handled using maximum likelihood estimation. To assess the validity of the model, diagnostic checks were conducted using dharma residual plots. If LMM assumptions were not satisfied, other appropriate statistical analyses were conducted (e.g., one-sided Wilcoxon tests). *Post-hoc* pairwise comparisons using estimated marginal means were conducted to assess differences in mean scores from pre- to post-intervention, pre- to 3-months post-intervention, and post- to 3-months post-intervention. All analyses were conducted using R Studio (Version 4.4.3), with LMMs fitted using the lme4 package, *post-hoc* comparisons using emmeans, model diagnostics using dharma, and data manipulation and visualization supported by the tidyverse package. Given the small sample size, all analyses were considered exploratory.

To analyze adoption, content analysis was used to identify common ideas based on participants' responses (72–74). Interview data was transcribed verbatim and coded by JG (female, OT, graduate student) on Microsoft Excel. Initial codes were generated from the data and iteratively refined through comparison across transcripts. Codes were then grouped into categories based on conceptual similarity and frequency. To enhance analytical rigor, a coding matrix was developed to compare responses across participant groups (participants with ABI and clinicians), allowing for systematic identification of shared and divergent perspectives. Throughout the analysis process, JS (female, OT, professor) provided regular input to review coding decisions, challenge interpretations, and refine category development. Representative quotations are presented to support identified categories.

Implementation data was compiled from fidelity checklists, completed by observers during the sessions. Researchers also reviewed video recordings of 30% of sessions to ensure the fidelity checklists were completed accurately and the sessions were delivered consistently. For consistency of delivery, the goal was having two people (facilitator and observer) present at 90% of sessions. For intervention attendance, the goal was to have all participants attend at least 80% of sessions. For session length and VR practice time, intervention consistency was determined by identifying the discrepancy between the session length and VR practice time; the goal was to be within a 20% difference. For session length, the aim was 80% of sessions at 30-minutes in length.

For maintenance, content analysis was used to analyze participants' answers to the questions asked at the 3-month follow-up. JG transcribed the responses from participants with ABI, while clinicians provided their responses electronically in written format. Data were coded inductively, with codes grouped into categories reflecting patterns over time (72). JS was consulted during the analysis. To enhance trustworthiness, we employed strategies during content analysis, such as involving multiple researchers in the analysis process to ensure divergent opinions regarding categorization of data and to ensure complementary perspectives (75). For example, codes, categories, and matrices were reviewed and refined by JS.

## Results

The following section outlines the study indicators within the RE-AIM framework, including who participated in the intervention (reach), the impact on the outcome measures (effectiveness), considerations for adoption (adoption), fidelity of the intervention (implementation), and if participants were willing to use the intervention long-term (maintenance).

### Reach

**Figure 2:**
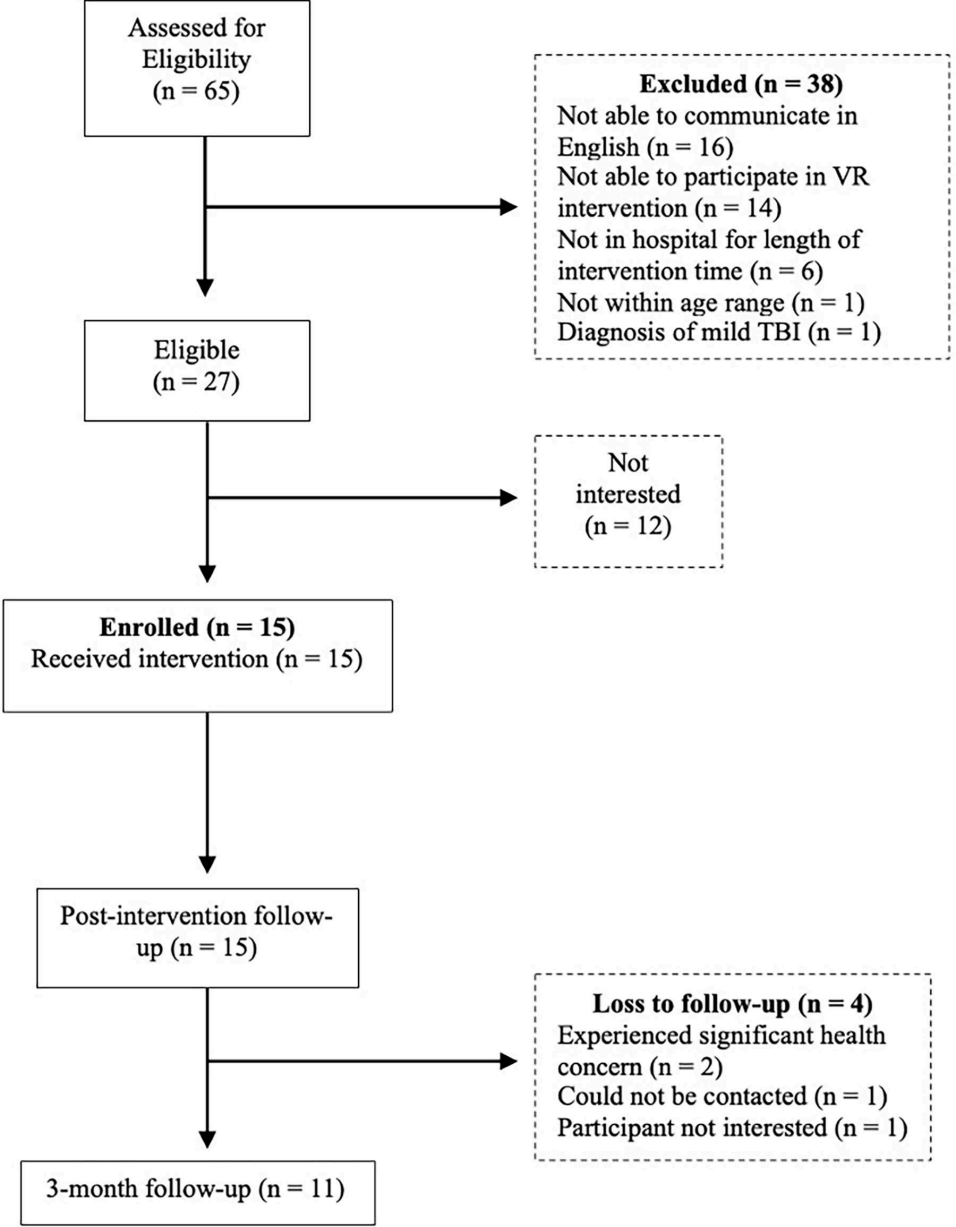
Participant flow chart.

We recruited participants from April 2024 to October 2024. 65 participants with ABI were screened of which 38 (58.5%) were not eligible due to varied reasons (e.g., not able to communicate in English, not able to participate in the VR intervention, etc.) and 27 (41.5%) were eligible (Figure 2). Of the eligible participants, 12 (44.4%) were not interested and 15 (55.6%) participants (mean age 46.5 years) were recruited for the study. Of the 15 participants (*n* = 5 females, *n* = 10 males), two had a severe TBI and 13 had moderate-to-severe stroke (Table 2). The mean days since injury were 125.5. Participants were receiving inpatient (*n* = 13) and outpatient (*n* = 2) therapy at GF Strong Rehabilitation Centre. Additionally, we invited nine OTs to facilitate sessions, this was the number of OTs working in the inpatient ABI rehabilitation and outpatient rehabilitation programs. Of the nine, five OTs (55.6%) (mean age 37.8 years) were recruited, two (22.2%) were interested but unable to facilitate a session due to scheduling conflicts, and two (22.2%) were not interested in participating. Three OTs worked in the inpatient ABI rehabilitation unit, one worked in the outpatient rehabilitation unit, and one worked as a clinical educator. On average they had 10.6 years of experience working as an OT and 7.7-years of experience working with the ABI population (Table 3). The OTs facilitated a total of seven sessions, with three OTs facilitating one session each and two OTs facilitating two sessions each. Research staff (trained by OTs) facilitated the remaining sessions. At the 3-month follow-up, 11 (73.3%) participants with ABI and all clinicians were successfully contacted. Four (26.7%) participants did not complete the 3-month outcome measures due to preference (*n* = 1), being unable to contact (*n* = 1), and experiencing significant health concerns (*n* = 2).

**Table 2:** Participants with ABI demographic table.

Characteristics	Total (*n* = 15)	Male (*n* = 10)	Female (*n* = 5)
Age, mean (SD)	46.5 (12.6)	45 (14.2)	50.4 (8.6)
Days since injury, mean (SD)	125.5 (131.3)	159.2 (141.5)	58.2 (26.0)
Type of injury, *n* (%)			
Stroke	13 (87)	8 (62)	5 (38)
Type of stroke, *n* (%)			
Ischemic	10 (77)	8 (80)	2 (20)
Left middle cerebral artery infarct	4 (40)	3 (75)	1 (25)
Right middle cerebral artery infarct	2 (20)	2 (100)	0 (0)
Left pontine infarct	2 (20)	1 (50)	1 (50)
Left lacunar infarct	1 (10)	1 (100)	0 (0)
Right cerebellar infarct	1 (10)	1 (100)	0 (0)
Hemorrhagic	3 (23)	0 (0)	3 (100)
Left thalamic hemorrhage	1 (33)	0 (0)	1 (100)
Left intracerebral hemorrhage	1 (33)	0 (0)	1 (100)
Aneurysmal subarachnoid hemorrhage	1 (33)	0 (0)	1 (100)
TBI	2 (13)	2 (13)	0 (0)
Cause of TBI, *n* (%)			
Motor vehicle accident	1 (50)	1 (50)	0 (0)
Construction site accident	1 (50)	1 (50)	0 (0)
Severity, *n* (%)			
Severe TBI (Glasglow Coma Scale)	2 (100)	2 (100)	0 (0)
Side impacted, *n* (%)			
Left	4 (20)	2 (50)	2 (50)
Right	8 (60)	6 (75)	2 (25)
N/A	3 (20)	2 (75)	1 (25)
Employment, *n* (%)			
Unemployed	5 (33)	5 (100)	0 (0)
On leave	4 (27)	1 (25)	3 (75)
Long-term disability	3 (20)	3 (100)	0 (0)
Short-term disability	2 (13)	0 (0)	2 (100)
Retired			
Education, *n* (%)	1 (7)	1 (100)	0 (0)
College	7 (47)	6 (86)	1 (14)
High school	3 (20)	2 (67)	1 (33)
University	2 (13)	0 (0)	2 (100)
Some university	2 (13)	2 (100)	0 (0)
Doctorate	1 (7)	0 (0)	1 (100)
Ethnicity, *n* (%)			
White/Caucasian	5 (33)	5 (100)	0 (0)
Southeast Asian	6 (40)	3 (50)	3 (50)
Indigenous	2 (13)	2 (100)	0 (0)
South Asian	1 (7)	0 (0)	1 (100)
Middle Eastern	1 (7)	0 (0)	1 (100)

Continuous variables are presented as mean [standard deviation (SD)]. Categorical variables are presented as frequency, *n* (%). Percentages for injury subtype categories (e.g., ischemic and hemorrhagic stroke) were calculated within the stroke subgroup. Percentages may not total 100 due to rounding.

**Table 3:** Clinician participants demographic table.

Characteristics	Clinician participants (*n* = 5)
Age, mean (SD)	37.8 (13.4)
Female, *n* (%)	5 (100)
Working environment, *n* (%)	
Inpatient rehabilitation	3 (60)
Outpatient rehabilitation	1 (20)
Educator role	1 (20)
Clinical experience (years), mean (SD)	10.6 (13.7)
ABI rehabilitation experience, mean (SD)	7.7 (9.9)
Ethnicity, *n* (%)	
White/Caucasian	4 (80)
Southeast Asian	1 (20)

Continuous variables are presented as mean [standard deviation (SD)]. Categorical variables are presented as frequency, *n* (%).

### Effectiveness

#### Primary outcome measure

LMM results showed a significant statistical improvement in NADL scores from pre- to post-intervention, b = 9.4, SE = 3.1, *t*(23.9) = 3.1, *p* = 0.005, 95% confidence interval (CI) [3.4, 15.4] (Table 4). Descriptive statistics for the primary outcome measure indicated that NADL scores increased from a baseline mean of 26.3 to a post-intervention mean of 35.7, with an observed mean change of 9.4 points, 95% CI [3.6, 15.2], and a large effect size (Cohen's *d* = 0.90). *Post-hoc* pairwise comparisons using estimated marginal means also revealed a significant statistical improvement in NADL scores from post- to 3-months post-intervention, *M* = 10.6, SE = 3.4, *t*(25.0) = 3.1, *p* = 0.005, 95% CI [3.5, 17.7] (Table 5). Descriptive data indicated that the mean NADL score increased from 37.8 at post-intervention to 48.0 at 3-month follow-up, with an observed mean change of 10.2 points, 95% CI [2.6, 17.8], and a large effect size (Cohen's *d* = 0.90). Diagnostic checks revealed residuals for NADL were normally distributed and homoscedastic (Supplementary Appendix E).

**Table 4:** Fixed effects table from LMM (pre- vs. post-intervention).

Outcome	Fixed effect estimate	SE	df	*t*	*p*	95% CI
NADL	9.4	3.1	23.9	3.1	0.005	[3.4, 15.4]
FMUE	4.9	1.6	24.0	3.1	0.005	[1.8, 8.0]
MOCA	2.1	0.6	23.7	3.4	0.002	[0.9, 3.4]
DLSES	4.4	2.6	24.2	1.7	0.104	[−0.7, 9.5]

**Table 5:** Estimated marginal means pairwise differences (post- vs. 3-months post-intervention).

Outcome	Marginal mean difference	SE	df	*t*	*p*	95% CI
NADL	10.6	3.4	25.0	3.1	0.005	[3.5, 17.7]
FMUE	4.5	1.8	24.2	2.5	0.02	[0.8, 8.2]
MOCA	−0.4	0.7	24.3	−0.5	0.625	[−1.8, 1.1]
DLSES	−0.6	2.9	24.3	−0.2	0.843	[−6.7, 5.5]

Exploratory subgroup analyses descriptively examined mean changes in NADL scores across participant characteristics. Greater mean improvements from pre- to post-intervention were observed in males compared with females, and in inpatients compared with outpatients (Table 6). Participants less than 3-months post-injury showed larger mean gains than those more than 3-months post-injury. People with TBI demonstrated greater mean improvement compared with those with stroke.

**Table 6:** Subgroup mean changes of primary outcome measure (pre-, post-intervention, 3-months post-intervention).

Subgroup	Pre- to post-intervention mean change	Post-intervention to 3 month post-intervention mean change
Diagnosis		
Stroke	8.3	10.2
TBI	16.5	N/A
Sex		
Male	11.1	4.7
Female	6.0	16.8
Care setting		
Inpatient	10.3	10.9
Outpatient	3.5	1.0
Time since injury		
<3-months post-injury	10.2	13.1
>3-months post-injury	8.0	−1.7

From post- to 3-months post-intervention, females demonstrated greater mean improvement than males, and inpatients showed larger gains compared with outpatients. Participants less than 3-months post-injury continued to show improvement, whereas those more than 3-months post-injury demonstrated a slight decline. Participants with stroke showed continued improvement; however, subgroup analysis for TBI could not be conducted at 3-months post-intervention due to incomplete follow-up. These findings are exploratory and should be interpreted with caution given the small subgroup sample sizes.

#### Secondary outcome measures

LMM results showed significant increase in FMUE scores from pre- to post-intervention, *p* = 0.005, 95% CI [1.8, 8.0] (Table 4). Pairwise contrasts using estimated marginal means for pre- to post-intervention and pre- to 3-months post-intervention are reported in Supplementary Appendix F. For post- to 3-months post-intervention, pairwise comparisons using estimated marginal means showed significant improvement in FMUE scores, *p* = 0.02, 95% CI [0.8, 8.2] (Table 5). LMM results showed significant improvements in MOCA scores from pre- to post-intervention, *p* = 0.002, 95% CI [0.9, 3.4] and pairwise comparisons revealed no significant improvement from post- to 3-months post-intervention, *p* = 0.625, 95% CI [−1.8, 1.1]. For DLSES, LMM showed no significant improvement from pre- to post-intervention, *p* = 0.104, 95% CI [−0.7, 9.5] and pairwise comparisons showed no significant improvements from post- to 3-months postintervention, *p* = 0.843, 95% CI [−6.7, 5.5]. Diagnostic checks revealed residuals for FMUE and MOCA were normally distributed and homoscedastic. For DLSES, diagnostic checks revealed residuals were homoscedastic but deviated significantly from normality (Kolmogorov–Smirnov test, *p* = 0.002) (Supplementary Appendix E). As a result, one-sided Wilcoxon tests were conducted and showed no significant improvements in DLSES scores from pre- to post-intervention (*V* = 63.5, *n* = 15, *p* = 0.255) and post- to 3-months post-intervention (*V* = 16.5, *n* = 11, *p* = 0.780).

### Adoption

Participants identified perceived benefits and challenges that affected adopting VR in clinical practice (clinicians) and in rehabilitation (people with ABI) (Tables 7, 8). Participants with ABI most commonly noted improvements in physical function as a benefit of VR, which may enhance their adoption of the device. Specifically, a participant with ABI stated that the ADLs in the VR device helped with upper extremity range of motion, noting “because of my limited range of motion with my arms, it was good for me… it improved my arm motions [by] making [dinner] and putting groceries away” (VRP15). Other perceived benefits that improved adoption included VR helping learn ADLs, stating “for example, the kitchen one was very knowledgeable on how to cook” (VRP08). Clinicians highlighted they may adopt VR into their daily practice as it could help participants after ABI and it was a good option to use complementary with OT. One clinician stated, “Maybe in addition to [therapy], [VR] would be great or if [participants] could do [VR] on the weekends when there's no therapist here” (OTP1).

**Table 7:** Perceived benefits of the Saebo-VR.

Perceived benefits	Total (*n* = 20)	Staff (*n* = 5)	Participants with ABI (*n* = 15)
Improving physical function (i.e., upper extremity function)	12	4	8
Improving cognition (i.e., memory, planning)	9	2	7
Learning ADLs	8	3	5
Enhancing visuospatial skills	5	3	2
Targeting ABI deficits	5	5	0
Increasing dose of therapy	5	5	0
Bringing diversity in rehabilitation activities	3	1	2
Motivating to use	3	3	0
Feeling like VR generally provides benefits	3	0	3

**Table 8:** Perceived challenges of the Saebo-VR.

Perceived benefits	Total (*n* = 20)	Staff (*n* = 5)	Participants with ABI (*n* = 15)
Technical issues	16	3	13
Negative impacts (e.g., pain)	8	1	7
Not being able to individualize VR treatment	11	5	6
Feeling like VR is generally not beneficial	10	7	3
Lack of variety of VR activities	9	1	8
Finding VR activities are not engaging	7	1	6
Increased reliance on compensatory movements	7	6	1
Lack of resources for implementation	5	5	0

Participants with ABI experienced negative impacts which impacted adoption of the VR, specifically there were instances where people experienced pain, fatigue, and stiffness in their upper extremity. A participant with ABI stated that the VR tasks warranted full movements of the impacted upper extremity which caused pain, noting “occasionally when the movement forced me to lift my whole arm, that's when it hurts for the shoulder” (VRP09). Other challenges that impacted adoption included not being able to individualize VR based on the participant's function, therefore the intervention was not applicable to all users. Some participants generally did not find VR beneficial, so they did not want to use it, noting “[VR] just doesn't really apply to me” (VRP01). Clinicians also highlighted a lack of varied ADLs in the VR device which led to boredom, “I felt like sometimes [participants] were bored or as if there weren't enough [activities] to try” (OTP1). Other clinicians noted barriers that impacted adoption, such as compensatory movements, reflecting “I was also kind of worried about [VR] reestablishing poor patterns and poor compensatory strategies because we're trying to get out of some of those things” (OTP3). All clinicians stated that there was a lack of resources for adoption of the VR device, including funding, time to learn the VR, staffing concerns, and training.

### Implementation

Participants attended 98.9% of sessions, which exceeded our goal of 80%. Of the attended sessions, 14 (93.3%) participants completed 12 VR sessions. One (6.7%) participant completed ten sessions and preferred not to complete the remaining two sessions. The mean session length was 25.8-minutes (SD = 2.1), and the mean VR practice duration was 20.1-minutes (SD = 2.2). The intervention consistency was not met as the discrepancy between the session length and actual VR practice time was more than a 20% difference. In terms of consistency of delivery, 95.5% of VR sessions had a facilitator and observer present and eight sessions did not have an observer present due to scheduling concerns, which exceeded our goal of 80%. A total of 84.3% of sessions were shortened, surpassing our goal of 80% of sessions being 30-minutes in length, due to various factors including scheduling reasons (61.3%), participant preference (16.7%), experiencing negative impacts (10.7%), facilitator choosing to end sessions (7.3%), VR technical issues (1.3%), and unspecified reasons (2.7%). Scheduling reasons included the session starting late and the participant not being able to participate for a full 30-minutes as they had an appointment afterwards or the facilitator ending the session 1- to 2-minutes early due to not having time for the VR device to load another activity. Video data indicated that all (100%) sessions were delivered consistently, with facilitators providing encouragement, assistance, breaks, and verbal cues.

### Maintenance

At the 3-month follow-up, most participants with ABI (*n* = 9, 60%) were willing to use the Saebo-VR device in rehabilitation, two (13.3%) were not willing to use the device, and four (26.7%) participants could not be contacted. Reasons why participants were willing to use the VR device included finding it helpful for improving function, convenient option for rehabilitation, and that VR provided an opportunity for additional rehabilitation. Reasons why participants were not willing to use the VR device included finding it boring and not helpful for enhancing function.

Regarding the training, four (80%) clinicians attended at least one of the two sessions that were scheduled. Most clinicians (*n* = 3, 75%) reported the training was sufficient and helpful for learning how to use the device, while one (25%) clinician felt the training was insufficient but valued the opportunity to gain hands-on experience with a patient population through participation in the study. Three (60%) clinicians indicated they would be willing to use the Saebo-VR clinically as an adjunct to conventional rehabilitation. They perceived that VR could increase the dose of therapy, noted that it is recommended in best practice guidelines for ABI rehabilitation, and believed it may be beneficial for certain participants. Two (40%) clinicians stated they would not use VR clinically as the device did not target specific skills or individualize treatment (i.e., fine motor skills), technical issues, time needed for implementation, and overall disinterest in VR.

## Discussion

Our study used the RE-AIM framework to understand the implementation of a VR intervention in clinical practice at a rehabilitation centre. Our results demonstrate strengths and challenges of implementing a VR intervention in clinical practice, as well as areas for improvement.

Our study identified several strengths associated with the clinical implementation of the VR intervention. Our findings demonstrated improvements in ADLs, upper extremity function, and cognitive performance following the intervention. In addition to these gains, participants perceived that VR enhanced their functional abilities, and supported learning and practicing ADLs in a meaningful way. The intervention was also viewed as a valuable complement to conventional occupational therapy, aligning with previous research showing that VR-based rehabilitation can promote task-specific practice, motivation, and functional recovery after neurological injury (47, 76). Together, these findings suggest that VR interventions can enhance clinical outcomes and patient engagement, supporting their integration into routine rehabilitation practice.

The VR intervention reached a small portion of people with ABI. Almost half of the screened participants were eligible for our study, however many declined to participate as they were not interested. After ABI, it is common for people to experience difficulties using technology, such as difficulties with using functions, feeling pressure from others when using technology, and not starting tasks if technology is involved (77). This may have been a reason to deter people from participating in our study despite being eligible. Further, after ABI, people often experience negative impacts including sensitivity to light, headaches, and nausea (78–80). VR may exacerbate these negative impacts, therefore discouraging people from participating in the study (81). People may also feel overwhelmed in a rehabilitation setting due to varied reasons (i.e., discharge disposition) or frustrated due to functional deficits, which may impact decision-making and discourage them from participating in research studies (82–85). Further, almost half of the screened participants were not eligible for our study. This may be due to VR devices lacking individualization or ability to grade activities; therefore, they may not match the needs and goals of people (86).

Our results suggest that the VR intervention was associated with improvements in functional abilities consistent with previous research (51, 87, 88). Improvements in ADL performance and upper extremity function were observed immediately after the intervention and at follow-up, indicating both immediate and sustained gains. Notably, while a previous study using a fully immersive VR system found no significant improvements in ADLs (i.e., NADL scores), the current study observed gains, suggesting that intervention design or level of immersion may influence outcomes (50). Consistent with this, other studies employing nonimmersive VR interventions that specifically incorporate ADL training have reported improvements in ADL measures (51, 52). Cognitive function improved immediately post-intervention but was not maintained at follow-up, while self-efficacy did not show significant change at any time point. It is important to consider that we cannot entirely attribute enhanced functional abilities to our VR intervention alone as this was a pre- and post-intervention study without a control group. VR may improve the dose of therapy by being used as an adjunct to therapy, therefore improvements in functional abilities may be attributed to people with ABI receiving an increased dose of therapy (i.e., usual occupational therapy and VR), similar to previous research (47). Clinicians may incorporate VR as an adjunct to conventional rehabilitation to potentially enhance therapy dose. VR can be used individually, with family support, or with rehabilitation assistants, and its use is supported by Canadian Stroke Best Practice recommendations to guide rehabilitation for ABI (89–91). VR may also encourage high repetitions of movements compared to conventional rehabilitation (92–94), and this could result in improvements in upper extremity range of motion. However, clinician participants reflected that the movements required by the VR device may establish inappropriate compensatory movements, which may result in pain, muscle contractures, misalignment of joints, and increased energy to complete movements (95). Therefore, it may be important for the VR to be used with supervision from a clinician to ensure repetitive movements establish effective movement patterns.

Interestingly, our results indicate that there were no significant improvements in self-efficacy for daily activities from pre- to post-intervention. Participating in VR can provide realtime feedback to participants (93, 96). Researchers have demonstrated that real-time feedback from VR paired with timely feedback and encouragement from clinicians may enhance participants' ability to understand their performance and improve their sense of achievement, which in turn could improve confidence in daily activities (97, 98). Although these characteristics are related to principles of self-efficacy and research has shown that VR may improve self-efficacy after stroke (99), these results did not align with the results in our study. This may be due to intervention-related factors, such as the dose at which our VR intervention was implemented, or it may be possible that the intervention did not translate to real-life performance of ADLs.

The exploratory subgroup findings, particularly those related to time since injury, should be interpreted with caution. Participants closer to the time of injury demonstrated larger improvements, which is consistent with established patterns of spontaneous neurological recovery in the early post-injury phase (100). Given the absence of a control group, it is not possible to attribute these differences to the intervention itself, and they likely reflect a combination of natural recovery processes and concurrent rehabilitation.

Our results also highlighted the intervention was delivered consistently; however, sessions were often shortened due to various reasons (i.e., scheduling conflicts). In inpatient and outpatient rehabilitation settings, people are often inundated with appointments throughout the day, and therefore participating in a research study may not have been a priority. Nevertheless, our study had consistent attendance for sessions, which is like other VR studies with the ABI population (100, 101). At times, engagement in rehabilitation can be of concern after ABI (102). People with ABI may be more interested in rehabilitation if they actively engage in the intervention and are motivated (103). Research shows using VR may improve motivation, therefore increased motivation coupled with high attendance can keep people with ABI engaged with rehabilitation, which could improve functional abilities (100, 104).

Although our results identified numerous perceived benefits of VR, participants highlighted key challenges for adoption, including technical issues and side effects (86, 105). Other challenges included the lack of individualization of the VR device based on participant factors, for example not being able to track fine motor movements (86). Previous research shows there is some benefit for upper extremity and ADL function by using VR that is customizable. For example, VR could have participant use both arms, therefore this may be an important consideration when designing VR interventions (Kim et al., 2016). Additionally, not having enough resources for implementation was identified as a barrier for adoption and maintenance, including cost and time to learn the equipment. These are common barriers identified by previous research, therefore, to successfully implement VR, there needs to be adequate funds for the equipment, and adequate time and training on how to use the equipment (86, 105). Previous research shows that assessing barriers before implementation and having a systematic plan for implementation based on a model may improve uptake of VR in clinical practice (86, 105).

Our results also showed a modest rate (i.e., 60%) of anticipated future use of the VR intervention. This may reflect the now discontinued Kinect platform (106). Although widely used in earlier rehabilitation research, Kinect-based systems are associated with reduced tracking fidelity, usability challenges, and complex setup, which may hinder sustained engagement and clinical integration (107, 108). In contrast, recent studies indicate that contemporary low-cost head-mounted systems offer improved tracking, ergonomics, and ease of use, largely addressing these earlier barriers (109). These technological advances may enhance feasibility and acceptability in clinical settings, suggesting that future implementations using modern head-mounted systems could achieve higher adoption and sustained use.

## Limitations

Our study had five main limitations. First, our study was a pre- and post-intervention without blinding, randomization, or a control group, making it difficult to determine whether observed improvements were due to the VR intervention or usual inpatient and outpatient rehabilitation. However, the RE-AIM framework allowed for data to be collected from different sources, which may improve the accuracy and relevance of the findings. Second, our sample only included two participants with TBI, so our results may be less applicable to the TBI population. Third, given the small sample size and associated risk of Type II error, the effectiveness data should be considered as preliminary, as the primary aim of this study was to understand implementation rather than efficacy. Fourth, at the 3-month follow-up, no participants were receiving the VR intervention. Therefore, while some outcome measures (e.g., NADL) showed significant improvements from post-intervention to follow-up, these changes cannot be attributed solely to the VR intervention, as no VR “dose” was delivered during this period and other rehabilitation or participation in ADLs may have contributed to the observed gains. Last, as we could not obtain four participants' data at the 3-month follow-up, we used LMMs to account for missing data. However, it is uncertain if data was missing at random which is an assumption of LMMs.

## Conclusion

Using a VR intervention in a clinical setting with the ABI population offers insight into the implementation of novel interventions and identifies strengths and challenges. Although VR shows promise for ABI rehabilitation, further multi-site research with larger samples can explore how VR interventions are integrated into ABI rehabilitation, providing deeper insight into the processes, challenges, and strategies for successful implementation in clinical settings. Importantly, our findings illustrate the importance of transitioning to more immersive platforms and demonstrate the critical role of implementation studies for identifying and addressing gaps between research and clinical practice.

## Data Availability

The raw data supporting the conclusions of this article will be made available by the authors, without undue reservation.
