# Supplementary material for: Virtual reality for activities of daily living rehabilitation after acquired brain injury
Source: Front Rehabil Sci. 2026 Jun 30;7:1726559. doi: 10.3389/fresc.2026.1726559 (PMC13365121; doi:10.3389/fresc.2026.1726559)
Supplement: Supplementary file 2 [file Supplementaryfile2.docx]

**Appendix B**

**Descriptions of the secondary outcome measures**

The Fugl-Myer Upper Extremity scale is performance-based measure of impairment. It is comprised of 33 items, which are scored on a scale of 0 to 2 (Singer & Garcia-Vega, 2017). For the purpose of this study, we assessed participants using specific subsections of the assessment, which is a common practice in previous research (Chen et al., 2022; Lin et al., 2021).

The Daily Living Self-Efficacy Scale is a 12-item scale measure that assesses self-efficacy for psychosocial functioning and self-efficacy for ADLs. The scoring varies from 0 (cannot do at all) to 100 (highly certain can do) (Maujean et al., 2014).

The Montreal Cognitive Assessment is a brief scale to screen for cognitive impairments in domains including, memory, visuospatial/executive function, language skills, orientation, attention, and delayed recall. Participants can get a maximum score of 30 (Nasreddine et al., 2005).

**Appendix C**

**Interview guide**

Interviewer notes:

- Encourage participants to share about their lived experiences

- Offer directed questions related to key constructs where appropriate, e.g., “When you talked about ____ would you say they were demonstrating acceptance of their situation?”

- General prompt ideas:

- “Can you talk about that more?”

- “Help me understand what you mean”

- “Can you give an example?”

**Welcome**

Thank you for having this Zoom call/telephone call to share your experiences of using virtual reality in rehabilitation.

We’re meeting here today for the study: The experience of using virtual reality in rehabilitation. The Principal Investigator of our study is Dr. Julia Schmidt.

We will go through a set of questions. Please provide your answers. If you do not feel comfortable answering, we can move on to the next question. The interview will take about 1-hour.

**Question guide:**

Participants

Q1. Can you describe your experience with virtual reality devices before participating in this study?
Q1a. Which devices have/do you use(d)?
Q1b. Were you comfortable using virtual reality devices before taking part in this study?

Q2. Can you describe your experience of using the Saebo-VR device?

Q3. What did you like about using the Saebo-VR device?

Q3a. Do you think these activities are reflective of everyday activities?

Q3b. How can these activities be improved?

Q3c. Do you think 30 minutes was an appropriate amount of time to do the activities?

Q4. What did you find difficult about using the Saebo-VR device?

Q4a. Did you experience any fatigue? Pain?

Q5. Would you take part in treatment that incorporates virtual reality devices again? Why or why not?

Q6. Would you continue to or begin using virtual reality devices in your personal life after this study? Why or why not?

Q7. Would you continue to or begin using virtual reality devices for your rehabilitation after this study? Why or why not

Q9. Do you have any ideas of how virtual reality devices can be better implemented in rehabilitation?

Q10. Any additional comments or concerns?

Clinicians

Q1. Can you describe your experience with virtual reality devices before participating in this study?
Q1a. Which devices have/do you use(d)?
Q1b. Were you comfortable using virtual reality devices before taking part in this study?

Q2. Can you describe your experience of implementing the Saebo-VR device with participants?

Q3. Did you feel well-trained on how to use the system before you implemented it with participants?

Q4. What did you like about implementing the Saebo-VR device?

Q5. What did you find difficult about implementing the Saebo-VR device?

Q6. Would you continue to implement the virtual reality in your clinical practice? Why or why not?

Q7. Do you foresee any barriers that would impact implementation of virtual reality in clinical practice?

Q8. Do you have any ideas of how virtual reality devices can be better implemented in rehabilitation?

Q9. Any additional comments or concerns?

**Appendix D**

**Sample Fidelity Checklist**

Checklist

Participant code:

Participant’s username: _________

Facilitator:

Observer:

Session #:

| **Structure of Session** | | **Check/Comm-ents** |
| --- | --- | --- |
| Getting Started | Facilitator introduced themselves to the participant (*or reminded participant who* facilitator *was*) (Y/N) |  |
|  | Facilitator introduced the VR equipment (*or asked the participant if they are ready to use the VR equipment*) **(**Y/N)  The participant was seated in front of the Saebo-VR (Y/N) (note: type of chair)  A login was created for the participant (*or* facilitator *logged in to the Saebo-VR using the participant’s username*) (Y/N)  Facilitator calibrated the participant’s upper limb movement using the Kinect camera (Y/N)  The participant used their L or R upper limb (circle one)  Session start time: |  |
| Sessions | The participant engaged in approximately 30-minutes of VR sessions (Y/N)   - The participant completed the balls and box task twice (time spent: ___)   The participant spent 20 minutes completing the VR task (actual time: ___)   - Indicate how many times the task was completed: ___ - Indicate difficulty level (motor and cognitive) of the tasks: ___   The participant remained seated during the VR sessions (Y/N)  The participant required a break(s) (Y/N)   - Number of breaks: ___ - Total minutes of break: ___   The VR equipment did not malfunction (Y/N) – provide details if malfunction occurred (i.e., type of malfunction, minutes it took to fix) |  |
| Manages Session | The sessions were adapted   - The session was shortened (actual time: ___) - The task was made more difficult (Y/N) - Facilitator provided ________ (number of) verbal cues during the session - The task was stopped (indicate time elapsed: ___)   Facilitator answered participant’s questions during the session  Facilitator encouraged participants during the session  The session ended on time  Reason why session ended early |  |
| Closing | No immediate adverse events were reported   - If reported, provide details: ________________   Facilitator thanked the participant for their time |  |
| General Comments | Number of times autocompletion function was used  Number of times facilitator provided physical assistance or completed the task for the participant  Names of tasks practiced  Other comments |  |

**Appendix E**

**Dharma plots**

*Dharma plot for NADL*


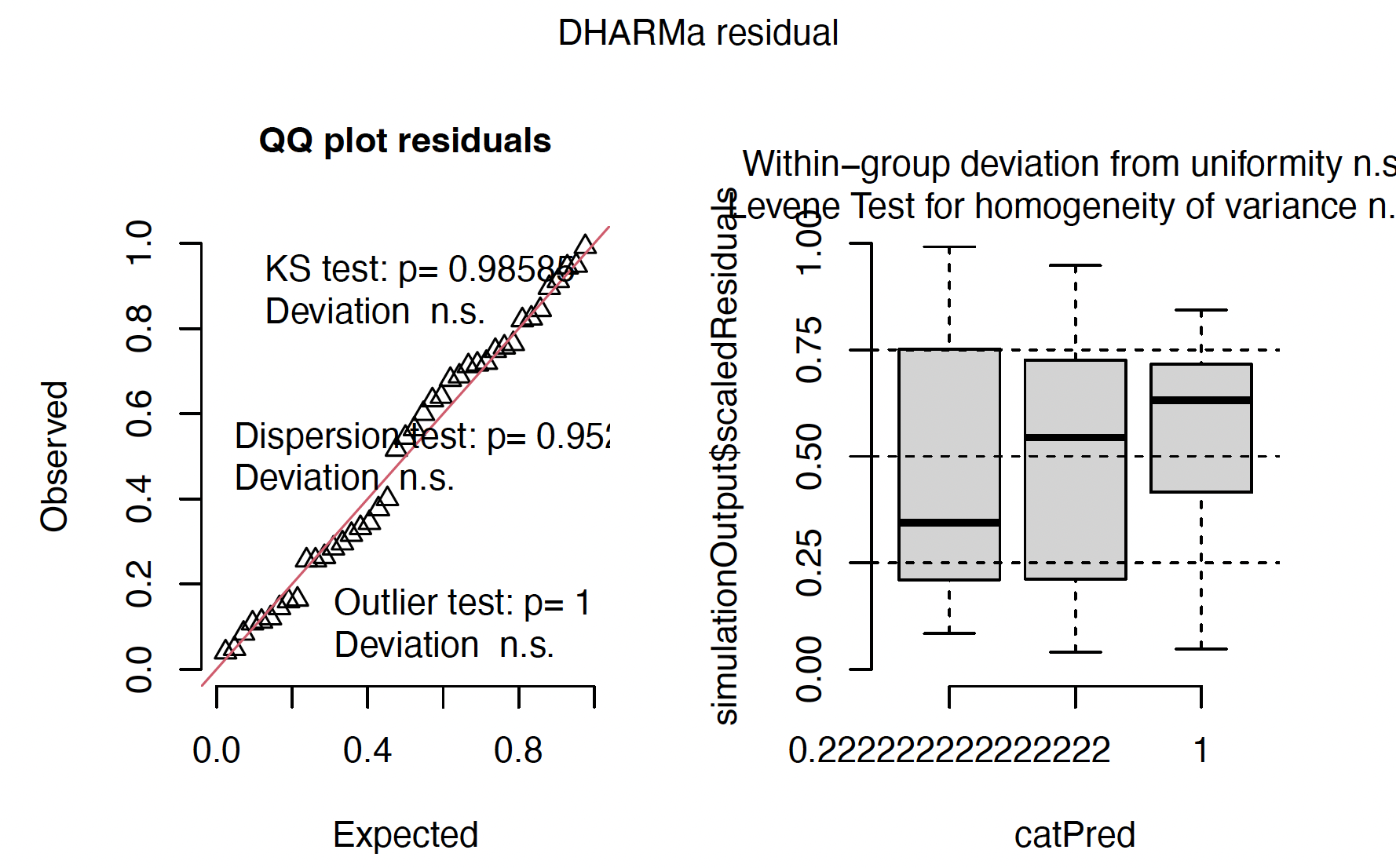


*Dharma plot for FMUE*


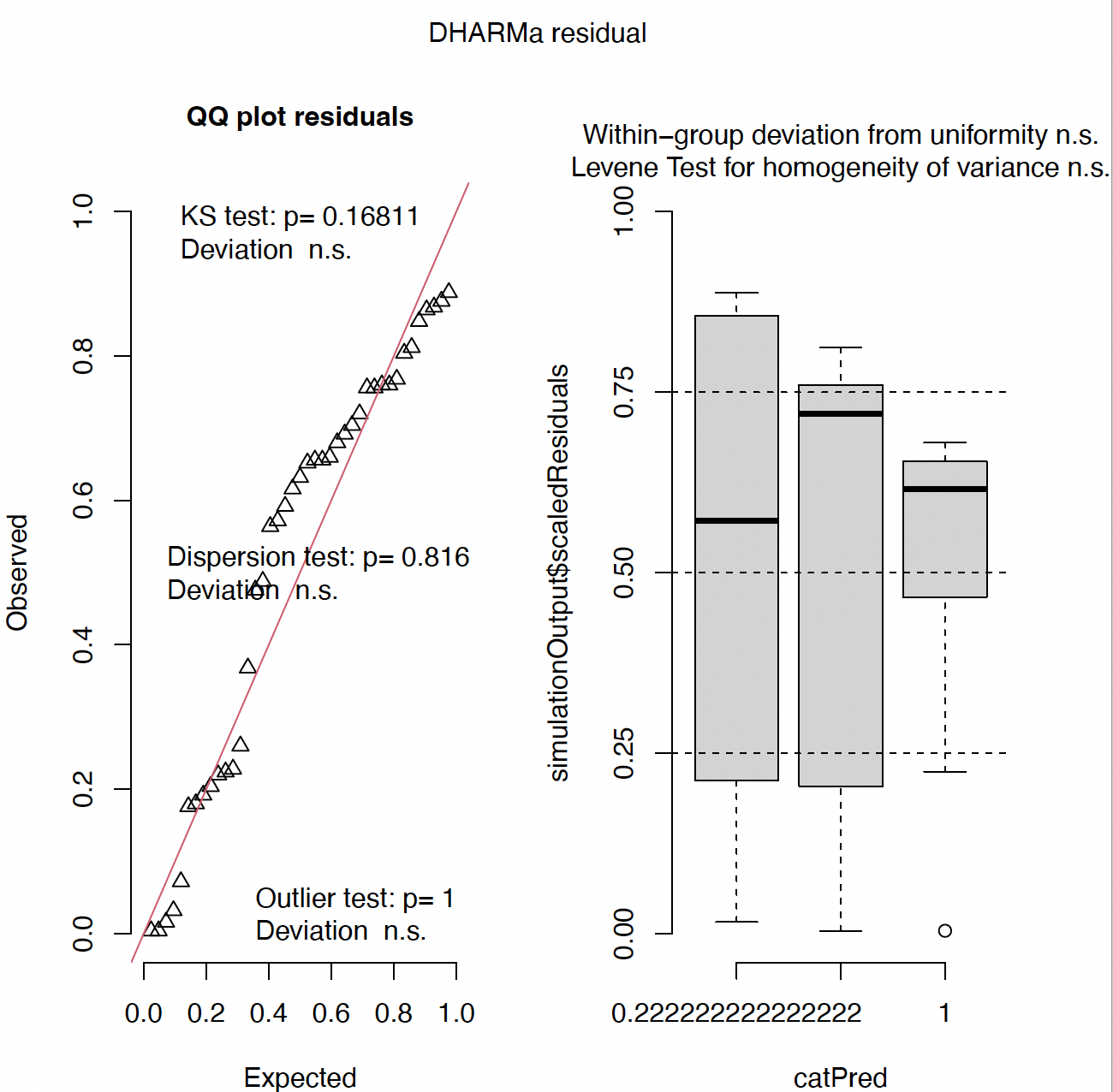


*Dharma plot for MOCA*


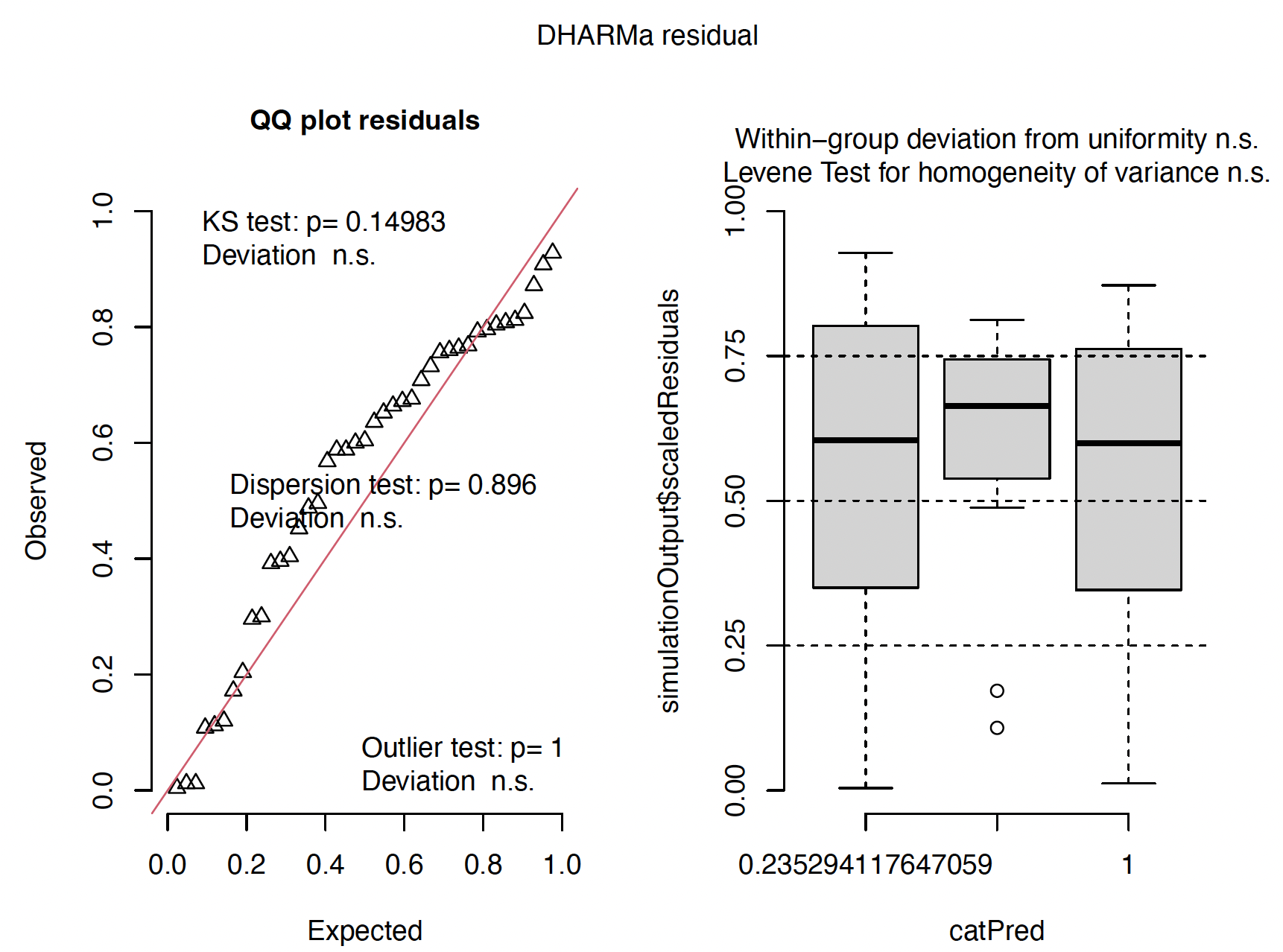


*Dharma plot for DLSES*


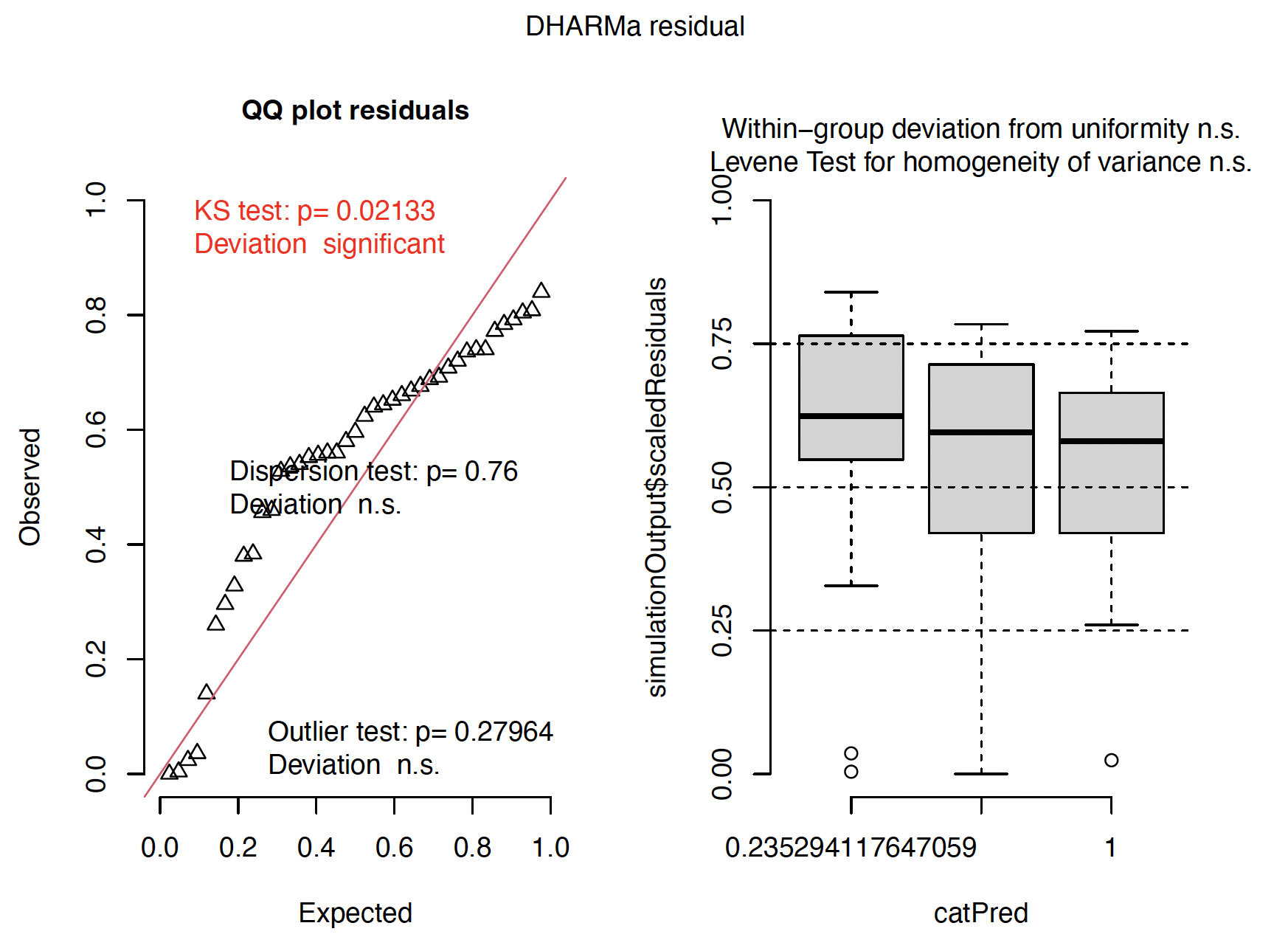


**Appendix F**

Table 1. Estimated marginal means pairwise differences (pre- vs. post-intervention)

| **Outcome** | **Marginal Mean Difference** | **SE** | **df** | **t** | **p** | **95% CI** |
| --- | --- | --- | --- | --- | --- | --- |
| NADL | 9.4 | 3.1 | 23.9 | 3.1 | 0.005 | [3.4, 15.4] |
| FMUE | 4.9 | 1.6 | 24.0 | 3.1 | 0.005 | [1.8, 8.0] |
| MOCA | 2.1 | 0.6 | 23.7 | 3.4 | 0.002 | [0.9, 3.4] |
| DLSES | 4.4 | 2.6 | 24.2 | 1.7 | 0.104 | [-0.7, 9.5] |

Table 2*.* Estimated marginal means pairwise differences (pre- vs. 3-months post-intervention)

| **Outcome** | **Marginal Mean Difference** | **SE** | **df** | **t** | **p** | **95% CI** |
| --- | --- | --- | --- | --- | --- | --- |
| NADL | 20.0 | 3.4 | 25.0 | 5.8 | <0.001 | [12.9, 21.1] |
| FMUE | 9.4 | 1.8 | 24.2 | 5.2 | <0.001 | [5.7, 13.1] |
| MOCA | 1.8 | 0.7 | 24.3 | -2.6 | 0.017 | [-1.9, 1.1] |
| DLSES | 3.8 | 2.9 | 24.3 | 1.3 | 0.205 | [-2.2, 9.9] |
